# Effects of physical therapy with neuromuscular electrical stimulation in acute and late septic shock patients: A randomised crossover clinical trial

**DOI:** 10.1371/journal.pone.0264068

**Published:** 2022-02-17

**Authors:** Alessandra Fabiane Lago, Anibal Basile-Filho, Anamaria Siriani de Oliveira, Hugo Celso Dutra de Souza, Daniele Oliveira dos Santos, Ada Clarice Gastaldi

**Affiliations:** 1 Department of Health Sciences, Ribeirão Preto Medical School, University of São Paulo, Ribeirão Preto, São Paulo, Brazil; 2 Division of Intensive Care Medicine, Department of Surgery and Anatomy, Ribeirão Preto Medical School, University of São Paulo, São Paulo, SP, Brazil; University of Texas Medical Branch at Galveston, UNITED STATES

## Abstract

**Background:**

Patients with sepsis and immobility in the intensive care unit are associated with muscle weakness, and early mobilisation can counteract it. However, during septic shock, mobilisation is often delayed due to the severity of the illness. Neuromuscular electrical stimulation (NMES) may be an alternative to mobilise these patients early. This study aims to identify whether NMES performed within the first 72 hours of septic shock diagnosis or later is safe from a metabolic perspective.

**Methods:**

This is the analysis of two randomised controlled crossover studies. Patients with acute septic shock (within the first 72 hours of diagnosis) and sepsis and septic shock in the late phase (after 72 hours of diagnosis) were eligible. Patients were submitted in a random order to the intervention protocol (dorsal decubitus position with the lower limbs raised and NMES) and control (dorsal decubitus position with the lower limbs raised without NMES). The patients were allocated in group 1 (intervention and control) or group 2 (control and intervention) with a wash-out period of 4 to 6 hours. Metabolic variables were evaluated by indirect calorimetry.

**Results:**

Sixteen patients were analysed in the acute septic shock study and 21 in the late sepsis/septic shock study. There were no significant differences between Oxygen Consumption (VO_2_) values in the acute phase of septic shock when the baseline period, intervention, and control protocols were compared (186.59 ± 46.10; 183.64 ± 41.39; 188.97 ± 44.88, p>0.05- expressed in mL/Kg/min). The same was observed when the VO_2_ values in the late phase were compared (224.22 ± 53.09; 226.20 ± 49.64; 226.79 ± 58.25, p>0.05). The other metabolic variables followed the same pattern, with no significant differences between the protocols. When metabolic variables were compared between acute to late phase, significant differences were observed (p<0.05).

**Conclusions:**

As metabolic rates in septic shock patients had no increase during NMES, either in the first 72 hours of diagnosis or later, NMES can be considered safe from a metabolic viewpoint, even despite the higher metabolic demand in the acute phase of shock.

**Trial registration:**

NCT03193164; NCT03815994. Registered on June 5, 2017; November 13, 2018 (clinicaltrials.gov/).

## Introduction

Septic shock is a life-threatening circulatory failure with inadequate tissue perfusion and altered metabolism. During septic shock, the metabolic rate increases, as evidenced by oxygen consumption which increases by 30% compared to normal basal metabolism [1]. Patients with sepsis and immobility in the intensive care unit (ICU) are associated with muscle weakness, which occurs rapidly within the first days of admission [2].

Early mobilisation applied to critically ill patients may improve muscle strength and functional status, as well as increases days alive and out of the hospital in the medium to long term [2]. Neuromuscular Electrical Stimulation (NMES) has been recognised as an alternative therapy to promote critical patient movement, especially to patients who cannot cooperate with physiotherapy due to sedation or decreased muscle strength [3].

Although some studies have found no difference when NMES was compared with usual care [4–6], Miao Liu et al. in 2020 reported in their meta-analysis results that NMES can improve muscle strength, shorten mechanical ventilation time, and decrease ICU and total length of stay [7]. Moreover, cross-sectional diameter, measured by muscle ultrasound, can be preserved in patients who received neuromuscular electrical stimulation [8–11]. Based on the beneficial results of NMES and scarce evidence supporting its safety in patients with septic shock [12], especially during the first hours of diagnosis, this study aimed to identify whether NMES performed within the first 72 hours of septic shock diagnosis and later is safe from a metabolic perspective.

## Methods

### Study design

The present study analyses two randomised controlled crossover studies (Trial registration: NCT03193164 and NCT03815994. Registered onJune 5, 2017 and November 13, 2018 https://clinicaltrials.gov/). The protocol was published previously [13]. The data were collected in an ICU of a Brazilian University Hospital from November 2018 to January 2020.

These studies followed the Consolidated Standards of Reporting Trials—extension to randomised crossover trials (CONSORT) [14] and were approved by the local ethics committee.

### Study participants and eligibility criteria

Two different populations were evaluated: septic shock patients in the acute phase (within the first 72 hours of diagnosis), and septic and septic shock patients in the late phase (after 72 hours of diagnosis). According to The Third International Consensus Definition for Sepsis and Septic Shock, sepsis was defined as a life-threatening organ dysfunction caused by a dysregulated host response to infection. Organ dysfunction was identified as an acute change in total SOFA score ≥2 points consequent to infection. Septic shock was identified as a clinical construct of sepsis with persisting hypotension requiring vasopressors to maintain MAP ≥65 mm Hg and having a serum lactate level >2 mmol/L despite adequate volume resuscitation [15–17].

Patients should have stable hemodynamic conditions after fluid resuscitation, vasoactive drugs, and mechanical ventilation. Concerning the population with sepsis and septic shock vasoactive drugs were not required.

Exclusion criteria were patients aged 18 years or less or over 85 years, pregnant women, those with brain death orneuromuscular diseases, or using a pre-existing neuromuscular blocker in the last 24 hours.

NMES use contraindications included fractures; burns; skin lesions; systemic vascular impairment diseases such as systemic lupus erythematosus, thromboembolic disease, deep vein thrombosis (which was not therapeutically anticoagulated for more than 36 hours); lower limb amputations; cardiac pacemaker; thrombocytopenia below 20,000/mm3; body mass index above 35 kg/m^2^; major lower extremity oedema; agitation; and/or signs of pain during the electrical stimulation.

Contraindications to begin or continue NMES procedure included the following: mean arterial blood pressure below 65 mm Hg; use of vasopressor >50% of the maximum dose (dopamine >12.5mg/kg per minute, vasopressin >0.02 U/min and norepinephrine >1mg/kg per minute); heart rate <50 or >140 bpm; arrhythmias with hemodynamic consequences; myocardial ischemia; temperature <34°C or >39°C; intracranial pressure >20 cmH_2_O; and 10% reduction in peripheral oxygen saturation (SpO_2_) baseline value or < 88% for more than 1 minute.

Contraindications to indirect calorimetry were the need for FiO_2_ > 0.6 and the presence of chest tubes.

### Recruitment organisation

All patients with a diagnosis of septic shock and sepsis were recruited at admission to the intensive care unit. An explanatory statement was given, and informed written consent was obtained before the commencement of the study. The author AFL had the final approval of a patient’s eligibility for the study. When patient relatives accepted the invitation to participate in the study, they signed the informed consent form.

### Randomisation and allocation

After the consent, AFL evaluated and determined the eligibility of patients. Afterwards, a nurse who was not involved in the assessment and interventions randomly allocated the participants in 1 of the 2 groups through simple randomisation (random numbers generated by the computer).

The allocation sequence was hidden by ABF through sequential numbered opaque sealed envelopes. After this step, AFL opened the envelope and started the protocol.

### Procedures

Patients were randomly submitted to the intervention protocol (dorsal decubitus position with limbs raised and NMES) and control (dorsal decubitus position with limbs raised without NMES). The patients were allocated in Group 1 (intervention and control) or group 2 (control and intervention) with a wash-out period of 4 to 6 hours.

### Outcomes

- Oxygen Consumption (VO_2_), Energy Expenditure (EE), Carbon Dioxide Production (VCO_2_), and Respiratory Quotient (RQ).

Indirect Calorimetry (IC) is a non-invasive method to measure heat content generated by the entire body according to substrate utilisation. The data provided by calorimetry are EE calculated from the amounts of VO_2_ and VCO_2_ through the respiratory gases.

Patients were submitted to IC during baseline, intervention and control protocols. The IC was measured by a portable calorimeter DELTATRAC II Metabolic Monitor (Datex-Ohmeda, Helsinki, Finland) connected to a mechanical ventilator (Evita XL, Dräger Medical, Lübeck, Germany) for 30 minutes in a stable condition without manipulation of the upper airways or changes in the ventilator settings. The ICU staff is trained in how to proceed when the IC is running. We considered as steady-state to be the point after 5 consecutive minutes measurement when oxygen consumption and carbon dioxide production varied by ±10%. This technique has already been employed in some previous studies [18–20] and validated [21]. The protocol was initiated after warming the calorimeter for 30 minutes. The gas and pressure (95% O_2_/5% CO_2_) were calibrated according to the manufacturer’s instructions.

The endothelial progenitor cells were collected, as described in the published protocol [13]. However, these cells were not visualised by flow cytometry, even though they had been analysed in two reputable laboratories.

### Protocol

The baseline period was defined as the initial moment without intervention.

Intervention protocol–dorsal decubitus position with the limbs raised and NMES. The patient was placed in a dorsal decubitus position on a bed with a headboard inclined at 30°, with lower limbs raised at 20°. Adhesive electrodes (90 x50 mm) were positioned in the gastrocnemius muscle, which.was cleared, and shaved when needed. A neuromuscular electrical stimulator Neurodyn II,Ibramed, Sao Paulo, Brazil) was used to provide symmetrical biphasic pulses of 250msec at 50Hz, 2 seconds ON (a 1-second rise time and a 1-second decay time), and 5 seconds of rest during 30 minutes at an intensity to generate visible muscle contractions and articular movements.

Control protocol–dorsal decubitus position with the limbs raised and without NMES. The patient was positioned as in the intervention protocol (headboard at 30°, dorsal decubitus position with lower limbs raised at 20° for 30 minutes).

An adverse event was defined as any change in mean arterial blood pressure (MAP) below 65mm Hg, heart rate <50 or >140 bpm, arrhythmias with hemodynamic consequences, myocardial ischemia, 10% reduction in SpO_2_ baseline value or <88%, and pain evaluated by the Brazilian version of the Behavioural Pain Rating Scale during NMES session [22].

### Statistical analysis

Data were summarised as mean and standard deviation for continuous variables, the median for ordinal variables, and frequencies and percentages for categorical variables. Data normality was verified by using the Shapiro-Wilk test. Differences between the protocols were assessed using analysis of variance ()ANOVA, by Friedman or Mann-Whitney tests. The p-value was used to check whether the protocol had an effect. The level of significance was set at 5%.

Statistical analysis was performed using Statistical Package for Social Sciences (SPSS), version 22.0 (IBM).

A new sample calculation was required to contemplate the metabolic variables. Based on Collings et al., who also evaluated VO_2_ by indirect calorimetry, 10 patients were needed. We have decided to interrupt our study with 16 and 21 septic patients (<72h) and sepsis/septic shock (>72h), respectively [23].

## Results

### Participant flow

**Fig 1:**
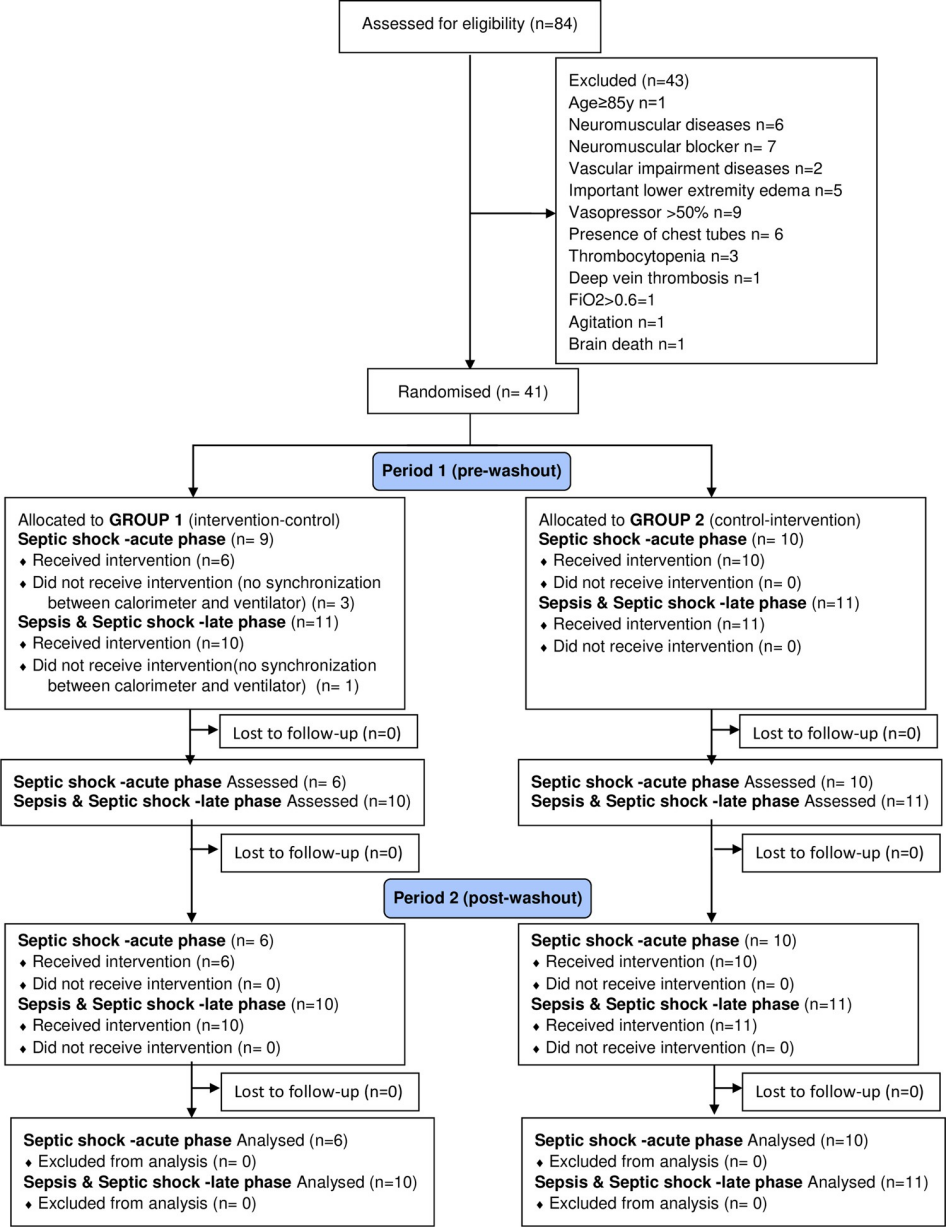
Participant flow. Group 1 (intervention—control) or group 2 (control—intervention) with a wash-out period of 4 to 6 hours.

Eighty-four patients were considered eligible for the study. Of these, 43 were excluded. Thus, a total of 41 patients were randomised, sixteen in septic shock within the first 72 hours of diagnosis and 21 in sepsis and septic shock in a period longer than 72 hours of diagnosis. After randomisation, in period 1 (before wash-out), 4 patients were excluded due to non-synchronisation between the calorimeter and mechanical ventilator. In period 2 (post-wash-out), no patient was excluded, and the study ended with 16 patients in septic shock < 72 hours and 21 patients in sepsis and septic shock > 72 hours. Fig 1 details the flow of patients.

### Demographic and clinical data

Table 1 describes the demographic and clinical data. Septic shock patients in the early phase (< 72 hours from the time of diagnosis) had a mean age of 56 ± 15.48 years old. Most of them were male (75%). In the late phase, the mean age was 57 ± 15.97, and most patients were also male (52%).

**Table 1:** Demographic and clinical data of patients.

Characteristic	Septic shock (Acute phase) n = 16	Sepsis and septic shock (late phase) n = 21
Age, yrs^a^	56 ± 15.48	57 ± 15.97
Male sex^b^	12 (75%)	11 (52%)
Admission diagnosis^b^		
Respiratory failure	2 (13%)	7 (33%)
Septic shock	5 (31%)	3 (14%)
Sepsis	0	3 (14%)
Neurologic disease	0	3 (14%)
Major surgery post-operative	6 (37%)	2 (10%)
Neoplasm	3 (19%)	2 (10%)
Exogenous intoxication	0	1 (5%)
Source of septic shock^b^		--
Abdominal	8 (50%)	
Pulmonary	4 (25%)	
Soft tissue	2 (13%)	
Urinary tract infection	1 (6%)	
Central nervous system	1 (6%)	
SAPS 3 score^a^	82.54 ± 19.21	75 ± 17.39
SOFA on day of intervention^a^	11 ± 3.17	8 ± 4.04
ICU mortality^b^	6 (38%)	5 (23%)
LOS ICU before intervention^c^	1,5 (1–2)	6 (1.5–8.5)
MV DAYS before intervention^c^	2 (1–3.5)	6.5 (3.5–9)
Use of corticosteroids^b^	5 (31%)	8 (38%)
Use of sedation on the day of Intervention^b^	15 (94%)	13 (62%)
Catecholamines^b^^,^^a^	16 (100%)	7 (33%)
Noradrenalin^b^^,^^a^ (μg/kg min)	16 (100%); 0.19 ± 0.16	7 (33%); 0.23 ± 0.20
Vasopressin^b^^,^^a^ (U/minute)	5 (31%); 16 ± 11.31	-
Nutrition;		
No nutrition^b^	10 (62%)	6 (29%)
Enteral^b^^,^^a^ (ml)	6 (38%); 1710 ± 295.98	15 (71%); 1326.67 ± 237.45
Harris-Benedict^a^	1476.12 ± 358.78	1420.70 ± 238.94
BMI^a^ Metabolic values^a^	28 ± 6.26	26 ± 5.57
^*^VO_**2**_(mL/kg/min) *baseline*	224.22 ± 53.09	186.59 ± 46.10
^*^VO_**2**_(mL/kg/min) *intervention*	226.20 ± 49.64	183.64 ± 41.39
^*^VO_**2**_(mL/kg/min) *control*	226.79 ± 58.25	188.97 ± 44.88
^*^EE (kcal/day) *baseline*	1482.85 ± 357.39	1265.66 ± 282.00
^*^EE (kcal/day) *intervention*	1488.58 ± 346.12	1243.58 ± 249.94
^*^EE (kcal/day) *control*	1492.43 ± 398.15	1274.95 ± 278.71
**VCO**_**2**_ (mL/kg/min) *baseline*	155.78 ± 41.59	149.88 ± 25.78
**VCO**_**2**_ (mL/kg/min) *intervention*	152.61 ± 45.10	144.97 ± 21.37
**VCO**_**2**_ (mL/kg/min) *control*	153.86 ± 48.86	148.10 ± 27.44
^*^^†^**RQ** *baseline*	0.70±0.05	0.82±0.15
^*^RQ *intervention*	0.68±0.05	0.82±0.15
^*^RQ *control*	0.68±0.06	0.80±0.15

SAPS: Simplified Acute Physiology Score; SOFA: Sepsis-related Organ Failure Assessment; LOS: Length of Stay; ICU: Intensive Care Unit; MV: Mechanical Ventilation; BMI: Body Mass Index; VO_2_- Oxygen consumption, EE- Energy Expenditure; VCO_2_-Carbon Dioxide Production; RQ: Respiratory Quotient.

^a^ Values expressed as mean ± SD

^b^ Values expressed as number (percentage)

^c^ Values expressed as median (interquartile range)

*p<0.005 comparisons between groups sepsis and septic shock -acute phase and septic shock—late phase.

^†^p<0.005 comparisons between RQ baseline and intervention in septic shock -acute phase.

All patients in the acute phase were diagnosed with septic shock. Some of them who had more than one diagnosis was categorised as major surgery post-operative (37%), only septic shock (31%), neoplasms (19%), and respiratory failure (13%). The focus of septic shock was abdominal (50%), pulmonary (25%), cutaneous (13%), as well as urinary and central nervous system (6% each). The main admission diagnoses of late-phase patients were a respiratory failure (33%), septic shock, sepsis and neurological diseases (14% each), post major surgery and neoplasm (10% each), and only in one patient (5%) exogenous intoxication.

The SAPS 3 score in acute patients was 82.54 ± 19.21, SOFA 11 ± 3.17, and ICU mortality 38%. On the other hand, in late-stage sepsis and septic shock patients, SAPS 3, SOFA, and ICU mortality were 75 ± 17.39, 8 ± 4.04, and 23%, respectively.

On the day of the intervention, median ICU stay and mechanical ventilation days for patients assessed in the first 72 hours of shock were 1.5 and 2, respectively, while for the remaining patients they were 6 and 6.5, respectively.

Thirty-one per cent of septic shock patients (<72h) were using corticosteroids, most (94%) on sedation and all receiving catecholamines. Unlike patients in sepsis and septic shock (>72h), 38% of the patients were in use of corticosteroids, 62% sedated, and only 33% in the use of noradrenaline. Table 1 displays the dosage of catecholamines.

Regarding the nutrition of septic shock patients in the acute phase, 62% were without nutrition on the day of the intervention and 38% on parenteral nutrition. Baseline energy expenditure and body mass index were on average 1476.12 ± 358.78 and 28 ± 6.26, respectively. As for the late-phase patients, a few (29%) had no nutrition, while the remaining received parenteral nutrition. For these, basal energy expenditure and body mass index were on average 1420.70 ± 238.94 and 26 ± 5.57, respectively.

### Metabolic data

**Fig 2:**
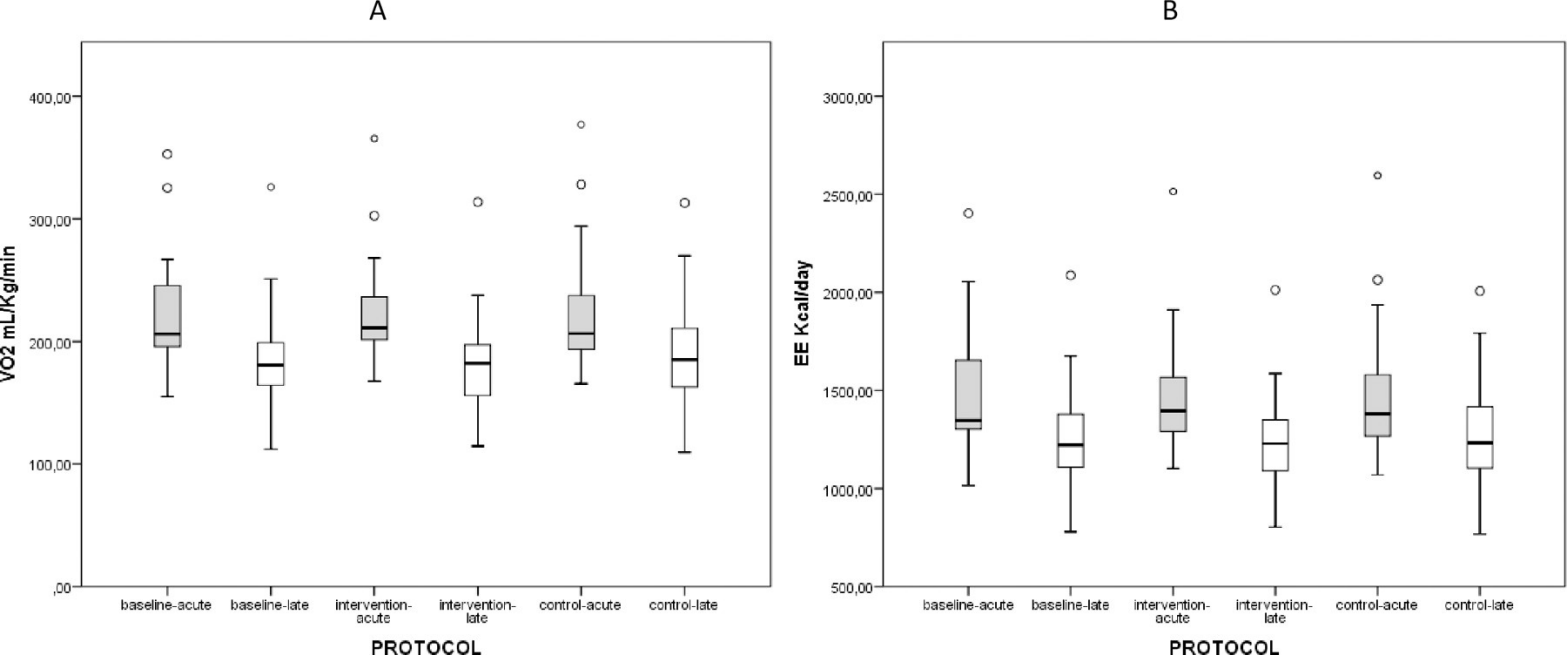
Oxygen consumption and energy expenditure during baseline, intervention, and control protocols in the acute and late phases. **A**. Oxygen consumption comparison between “septic shock acute phase” and “sepsis and septic shock late phase (p<0.05). Comparison among baseline, intervention and control protocols (p>0.05). **B**. Energy expenditure comparison between “septic shock -acute phase” and “sepsis and septic shock -late phase” (p<0.05). Comparison among baseline, intervention and control protocols (p>0.05). Grey boxes- the acute phase. White boxes- the late phase.

Table 1, Fig 2, and S1 and S2 Tables display the results of the metabolic variables. Patients with septic shock (< 72 h) and in sepsis and septic shock (>72h) did not show significant differences when compared intragroup for oxygen consumption, energy expenditure, carbon dioxide production, and respiratory quotient (i.e., among resting, intervention, and control protocols). Otherwise, respiratory quotient values in septic shock patients (<72h) showed statistical differences, but without clinical relevance when comparing the resting to intervention protocols (0.70±0.05; 0.68±0.05, p<0.005).

When intergroup comparison was made (i.e., comparing patients in septic shock <72h with those in sepsis and septic shock >72 hours), oxygen consumption and energy expenditure showed significantly higher values and, in all protocols (baseline, intervention and control), patients diagnosed in the acute phase were observed. Lower RQ values were observed in septic shock patients in the acute phase (Table 1).

Only one patient demonstrated pain during the NMES, which was attenuated when the intensity was decreased.

## Discussion

This study did not show significant metabolic changes when NMES was applied in the acute and late phase of septic shock patients. Early mobilisation is often delayed given the severity of the disease the patient suffers from. NMES may be a safe alternative to muscle contraction and mobilisation due to the low metabolic demand.

Hickmann et al. studied 21 septic shock patients and found that manual mobilisation and passive/active cycling therapy are safe and preserve muscle fibre [24]. Nevertheless, the safety of an intervention is only assured when different evaluations are conducted. In this context, metabolic, haemodynamic, ventilatory, inflammatory, tissue, and muscle assessments may indicate the security of an intervention. In this study, we collaborate in part with this long investigation by demonstrating the low metabolic cost of NMES. Therefore, it is a safe intervention in patients with acute septic shock from a metabolic point of view.

We also demonstrated that the acuteness of illness should be considered. The baseline metabolic rates were significantly higher in patients with acute septic shock than in those in the late phase. These results are consistent with the flow phase described by Cuthbertson in 1942 [25]. An ebb phase (1–2 days) starts immediately after a traumatic shock, followed by a flow phase (e.g., 3 to 10 days). A decrease in metabolic rate characterises the ebb phase. The opposite happens in the flow phase, i.e., increased metabolic rate, followed by a gradual decline [25, 26].

Little is known about the effects of NMES on septic shock patients within the first 72h. A Danish group investigated the effects of electrical stimulation on muscle trophism in patients with acute septic shock and found a significant decrease in quadriceps volume as early as the first week after admission. However, this loss of muscle mass was not counterbalanced by electrical stimulation. Nevertheless, it is worth saying that the authors evaluated only a single leg in eight men [12].

On the other hand, the effects of NMS on septic patients have been a little further explored. Som studies have demonstrated that the benefits might be beyond only muscle strength increase. NMES has been reported to improve muscle microcirculation and even to restore endothelial function [27, 28].

In addition to these specific studies on sepsis, a systematic review with meta-analysis has not shown differences between NMES plus usual care and usual care alone in terms of muscle strength, ICU mortality, MV duration, or ICU stay length. Although some of the patients analysed were diagnosed with sepsis or septic shock, we cannot say whether the outcomes were better or worse. NMES meta-analysis is generally affected by the high heterogeneity of measurements and methods with different muscle groups and device settings [6].

Likewise, Fossat et al. conducted an elegant trial enrolling critically ill adult patients, among which some septic and non-septic. The authors applied an in-bed leg cycling plus electrical stimulation. The stimulation group did not differ from the usual care although muscle strength by MRC score may have achieved a ceiling effect [4]. Furthermore, the usual care was a progressive multistep program adapted from the well-established program described and used by Schweickert et al. [29], whereas NMES was delivered only in in-bed cycling, which may have reduced the neuromuscular efficacy.

Our study has some limitations. The first is the difficulty in recruiting stable septic shock patients with an adequate dosage of vasopressors and within the first 72 hours of septic shock diagnosis. Given this fact and due to COVID-19 pandemic of, we decided to end the study. The second is that we planned to analyse endothelial progenitor cells, but we could reproduce the cell analysis-based method of Stefanou et al. [28].

Based on this finding, we could collaborate with exercise prescription decision making. The principle “one size does not fit all” has to be taken into consideration. Increased demand for physical activities in the acute septic shock phase seems to be unreasonable, as they already have high metabolic rates. Rehabilitation prescriptions for critically ill patients must be individually tailored according to illness, acuteness, and limitations.

As this study has only a small part of safety evaluations, further mobilisation-related investigations in septic shock patients during the first days of diagnosis are still needed to help clinicians mobilise these patients safely as soon as possible.

## Conclusions

A further increase in metabolic demand is not desirable during the acute phase of septic shock. As there were no metabolic changes during NMES, we can conclude that NMES in patients with septic shock, either within the first 72 hours of diagnosis or later, is safe in a metabolic view.

## Supporting information

S1 Checklist CONSORT 2010 checklist of information to include when reporting a randomised trial*. (DOC)

S1 Table Metabolic variables in septic shock patients in the acute phase. VO_2_- Oxygen consumption, EE- Energy Expenditure; VCO_2_-Carbon Dioxide Production; RQ: Respiratory Quotient.
(DOCX)

S2 Table Metabolic variables for septic shock and septic patients in the late phase. VO_2_- Oxygen consumption, EE- Energy Expenditure; VCO_2_-Carbon Dioxide Production; RQ: Respiratory Quotient.
(DOCX)

S1 File (DOCX)
