# Supplementary material for: Effects of physical therapy with neuromuscular electrical stimulation in acute and late septic shock patients: A randomised crossover clinical trial
Source: PLoS One. 2022 Feb 17;17(2):e0264068. doi: 10.1371/journal.pone.0264068 (PMC8853464; doi:10.1371/journal.pone.0264068)
Supplement: S1 Table — VO2- Oxygen consumption, EE- Energy Expenditure; VCO2-Carbon Dioxide Production; RQ: Respiratory Quotient. (DOCX) [file pone.0264068.s002.docx]

**SUPPORTING INFORMATION**

**S1Table. Metabolic variables in septic shock patients in the acute phase.**

| PATIENT | VO2  Baseline | EE  Baseline | VCO2  Baseline | RQ  Baseline | VO2  Intervention | EE  Intervention | VCO2  Intervention | RQ  Intervention | VO2  Control | EE  Control | VCO2  Control | RQ  Control |
| --- | --- | --- | --- | --- | --- | --- | --- | --- | --- | --- | --- | --- |
| 1 | 221 | 1470 | 159 | 0.72 | 219 | 1454 | 155 | 0.7 | 240 | 1591 | 167 | 0.69 |
| 2 | 207.27 | 1340.17 | 128.64 | 0.62 | 203.68 | 1307.66 | 121.35 | 0.59 | 201.16 | 1299.61 | 124.64 | 0.62 |
| 3 | 169.72 | 1118.28 | 119.96 | 0.7 | 167.53 | 1103.15 | 118.06 | 0.7 | 171.51 | 1121.57 | 116.14 | 0.67 |
| 4 | 194.87 | 1290.23 | 139.1 | 0.71 | 207.23 | 1353.66 | 136.43 | 0.65 | 195.5 | 1282.42 | 132.59 | 0.67 |
| 5 | 196.89 | 1338 | 159.92 | 0.81 | 216.8 | 1456.8 | 165.23 | 0.75 | 208.24 | 1409.85 | 165.36 | 0.79 |
| 6 | 352.8 | 2404.09 | 278.53 | 0.78 | 365.59 | 2514.76 | 301.53 | 0.82 | 376.98 | 2597.04 | 312.75 | 0.82 |
| 7 | 267 | 1788 | 189 | 0.71 | 268 | 1784 | 182 | 0.66 | 294 | 1937 | 190 | 0.65 |
| 8 | 211 | 1398 | 142 | 0.68 | 215 | 1423 | 143 | 0.67 | 205 | 1370 | 144 | 0.71 |
| 9 | 235.97 | 1603.77 | 188.85 | 0.79 | 230.17 | 1515.38 | 156.66 | 0.67 | 215.47 | 1410.47 | 143.01 | 0.66 |
| 10 | 255,11 | 1703 | 185.34 | 0.72 | 242.19 | 1617.9 | 177.39 | 0.72 | 234.9 | 1569.25 | 172.48 | 0.73 |
| 11 | 205.13 | 1331.8 | 130.6 | 0.63 | 196.9 | 1278.8 | 126.1 | 0.63 | 217.57 | 1393.09 | 126.54 | 0.57 |
| 12 | 154.95 | 1014.38 | 106.91 | 0.68 | 174.76 | 1143.9 | 118.69 | 0.68 | 165.6 | 1069.52 | 104.98 | 0.63 |
| 13 | 200.76 | 1314.38 | 134.38 | 0.67 | 200.5 | 1301.57 | 127.89 | 0.63 | 191.58 | 1253.09 | 128.13 | 0.66 |
| 14 | 325.24 | 2054.35 | 165.8 | 0.5 | 302.62 | 1911.42 | 155.24 | 0.5 | 328.05 | 2064 | 162.44 | 0.49 |
| 15 | 186.62 | 1202.3 | 114.82 | 0.61 | 202.1 | 1283.04 | 112.3 | 0.55 | 183.08 | 1177.4 | 111.78 | 0.6 |
| 16 | 203.21 | 1355 | 149.76 | 0.73 | 207.18 | 1368.28 | 144.96 | 0.69 | 200.04 | 1333.71 | 147.65 | 0.73 |

VO_2_- Oxygen consumption, EE- Energy Expenditure; VCO_2_-Carbon Dioxide Production; RQ: Respiratory Quotient.
