# Supplementary material for: Effects of physical therapy with neuromuscular electrical stimulation in acute and late septic shock patients: A randomised crossover clinical trial
Source: PLoS One. 2022 Feb 17;17(2):e0264068. doi: 10.1371/journal.pone.0264068 (PMC8853464; doi:10.1371/journal.pone.0264068)
Supplement: S2 Table — VO2- Oxygen consumption, EE- Energy Expenditure; VCO2-Carbon Dioxide Production; RQ: Respiratory Quotient. (DOCX) [file pone.0264068.s003.docx]

**S2 Table. Metabolic variables for septic shock and septic patients in the late phase.**

| PATIENT | VO2  Baseline | EE  Baseline | VCO2  Baseline | RQ  Baseline | VO2  Intervention | EE  Intervention | VCO2  Intervention | RQ  Intervention | VO2  Control | EE  Control | VCO2  Control | RQ  Control |
| --- | --- | --- | --- | --- | --- | --- | --- | --- | --- | --- | --- | --- |
| 1 | 112.08 | 779.61 | 107.59 | 0.96 | 114.55 | 802.8 | 113.06 | 0.99 | 109.64 | 768 | 109.64 | 0.99 |
| 2 | 326 | 2087 | 181 | 0.55 | 313.8 | 2013 | 178.04 | 0.56 | 313.05 | 2007.09 | 177.14 | 0.56 |
| 3 | 195 | 1292 | 137 | 0.71 | 209.5 | 1367 | 136.9 | 0.66 | 194 | 1265 | 127.46 | 0.66 |
| 4 | 223.19 | 1437.38 | 134.18 | 0.6 | 208.25 | 1350 | 131.15 | 0.63 | 219.54 | 1415.86 | 133.32 | 0.6 |
| 5 | 167.5 | 1090 | 101.64 | 0.62 | 169.21 | 1090.37 | 105.64 | 0.62 | 167.4 | 1056 | 91.96 | 0.54 |
| 6 | 187.19 | 1235.52 | 131.99 | 0.73 | 193.33 | 1317.61 | 159.47 | 0.83 | 159.63 | 1092.04 | 136.65 | 0.86 |
| 7 | 139.41 | 985.61 | 139.1 | 0.99 | 151.14 | 1066.47 | 148.61 | 0.98 | 142.81 | 987.54 | 125.54 | 0.9 |
| 8 | 250.91 | 1675.19 | 182.71 | 0.72 | 237.59 | 1587.3 | 127.32 | 0.72 | 269.87 | 1792.66 | 190.22 | 0.7 |
| 9 | 180.75 | 1221.09 | 143.81 | 0.8 | 188.07 | 1280.66 | 154.88 | 0.84 | 173.47 | 1173.25 | 139.45 | 0.82 |
| 10 | 133.02 | 899.9 | 110.52 | 0.82 | 143.52 | 959.42 | 111.52 | 0.77 | 144.8 | 958.95 | 107.22 | 0.73 |
| 11 | 230.36 | 1526 | 162.5 | 0.7 | 195.84 | 1284.48 | 132.72 | 0.67 | 217.06 | 1450.44 | 160.77 | 0.74 |
| 12 | 175.4 | 1174.23 | 133.98 | 0.76 | 170.59 | 1142 | 131.1 | 0.76 | 195.55 | 1278.47 | 130.23 | 0.72 |
| 13 | 191.6 | 1337.57 | 176.2 | 0.96 | 177.36 | 1229 | 159.23 | 0.89 | 210.78 | 1444.9 | 177.24 | 0.84 |
| 14 | 149.73 | 1093.94 | 168.64 | 1.14 | 155.92 | 1138.66 | 174.71 | 1.12 | 150.15 | 1104.11 | 173.06 | 1.15 |
| 15 | 153.4 | 1108.38 | 165.36 | 1.07 | 156.42 | 1125.36 | 165.57 | 1.05 | 162.79 | 1161.95 | 166.54 | 1.02 |
| 16 | 165 | 1148.14 | 151.59 | 0.91 | 145.4 | 1033.57 | 147.9 | 1.02 | 171.43 | 1188.35 | 154.47 | 0.89 |
| 17 | 191 | 1331 | 164 | 0.86 | 182 | 1262 | 154 | 0.85 | 194 | 1356 | 167 | 0.86 |
| 18 | 164.24 | 1148.3 | 154.24 | 0.93 | 151.84 | 1046.86 | 135.31 | 0.89 | 166.31 | 1147.72 | 147.34 | 0.88 |
| 19 | 172.25 | 1155.14 | 132.95 | 0.76 | 182.32 | 1216.47 | 136.31 | 0.74 | 185.21 | 1232.38 | 136.4 | 0.73 |
| 20 | 199.06 | 1378.7 | 176.26 | 0.88 | 212.75 | 1452.68 | 175.5 | 0.82 | 219.43 | 1500 | 181.9 | 0.82 |
| 21 | 211.3 | 1474.16 | 192.33 | 0.9 | 197.18 | 1349.58 | 165.6 | 0.83 | 201.59 | 1393.41 | 176.71 | 0.87 |

VO_2_- Oxygen consumption, EE- Energy Expenditure; VCO_2_-Carbon Dioxide Production; RQ: Respiratory Quotient.
