# Supplementary material for: Effects of physical therapy with neuromuscular electrical stimulation in acute and late septic shock patients: A randomised crossover clinical trial
Source: PLoS One. 2022 Feb 17;17(2):e0264068. doi: 10.1371/journal.pone.0264068 (PMC8853464; doi:10.1371/journal.pone.0264068)
Supplement: S1 File — (DOCX) [file pone.0264068.s004.docx]

**THE MAIN POINTS OF THE ORIGINAL PROTOCOL ( submitted to and approved by our ethics committee).**

**Title:** Effects of physiotherapy with neuromuscular electrical stimulation in patients with septic shock.

**Decision number:** 1.887.272 version 3.0 12 July 2018

**OBJECTIVE**

The aim of this study is to evaluate the effects of physiotherapy with neuromuscular electrical stimulation in patients with septic shock.

**METHODS**

**Study design**

It is a randomised, controlled, cross-over study.

**Eligibility**

Patients admitted to the adult ICU in the first 72 hours after septic shock diagnosis will be eligible for this study.

Exclusion criteria were patients aged 18 years or less or over 85 years, pregnant women, brain death, neuromuscular diseases, or the use of a pre-existing neuromuscular blocker in the last 24 hours.

Contraindications for the use of NMES included fractures, burns, skin lesions, systemic vascular impairment diseases such as systemic lupus erythematosus, thromboembolic disease, deep vein thrombosis (which was not therapeutically anticoagulated for more than 36 hours), lower limb amputations, cardiac pacemaker, thrombocytopenia less than 20,000/mm3, body mass index greater than 35 kg/m2, important lower extremity edema, agitation, and/or signs of pain during the electrical stimulation.

Contraindications to begin or continue NMES procedure included the following: mean arterial blood pressure less than 65 mm Hg, use of vasopressor >50% of the maximum dose (dopamine >12.5mg/kg per minute; vasopressin >0.02 U/min and norepinephrine >1mg/kg per minute), heart rate <50 or>140 bpm, arrhythmias with hemodynamic consequences, myocardial ischemia, temperature <34ºC or >39ºC, intracranial pressure >20 cmH2O, decrease in 10% peripheral oxygen saturation (SpO2) baseline value or <88% for more than 1 minute.

Contraindications to indirect calorimetry: the need for FiO2 > 0.6, PEEP< 12 cmH2O and presence of chest tubes.

**Protocol**

The Informed Consent Form will be requested from the patient's next of kin. The patients will be randomly allocated to the sequence of sessions through sequential numbers kept in opaque, non-translucent envelopes, closed until the beginning of each session. The study will be divided into two phases, the first phase to be performed within the first 72 hours of septic shock and the second phase to be performed 3 days after the first evaluation.

The patients will be submitted, in random order, to the intervention protocol (NMES) and control (positioning). Thus, they could be allocated to group 1 (NMES and control) or group 2 (control and NMES). Both groups' measurements will be performed in the initial and final period, with a wash-out period of 4 to 6 hours between them.

*Intervention protocol* – dorsal decubitus position with the limbs raised and NMES. The patient was positioned on a headboard at 30° in the dorsal decubitus position with the limbs raised to 20°. The location of the electrical current was cleared, and trichotomy was performed when necessary. Adhesive electrodes 90 x50mm was positioned in the gastrocnemius. The stimulator device was the Neurodyn II (Ibramed, Sao Paulo, Brazil) to provide symmetrical biphasic pulses of 50Hz, 250msec pulse duration, 2 seconds on (1 second of rise time and 1 second of time of decay), and 5 seconds of rest during 30 minutes at an intensity capable of generating visible contractions and articular motion.

During the session, pain will be assessed through the Brazilian version of the Behavioral Pain Rating Scale (BPS), with the aim of measuring pain experiences in patients unable to communicate verbally. Since they will be unconscious and on mechanical ventilation, i.e. unable to express themselves verbally effectively, either due to the underlying disease or the use of sedatives (Morete et al., 2014).

*Control-Positioning Protocol-* The patient will be positioned in dorsal decubitus, head at 30º, with the lower limbs elevated at 30º for 30 minutes.

**Measurements**

*Indirect calorimetry*

The patients will be submitted to energy expenditure and oxygen consumption measurements during the protocols mentioned above. Gas and pressure calibration will be performed according to the manufacturer's instructions and prior to the measurement. After this step, the calorimeter will be connected to the mechanical ventilator and then the measurements will be performed. Indirect calorimetry will be performed using a calorimeter (DELTATRAC II Metabolic Monitor; Datex-Ohmeda, Helsinki, Finland)

*Blood Sample and Flow Cytometry Analysis*

Blood samples will be collected from a central venous access for evaluation of endothelial progenitor cells before and after physiotherapy sessions. The protocol for the quantification of different subpopulations of EPC will be performed as described by Duda et al., 2007 and Fadini, Losordo and Dimmeler 2011. The collected blood will be discarded after the analysis.

*Ultrasonography*

Ultrasonography will allow adjusting the positioning of the electrodes, adjusting the intensity and assessing muscle contraction in real time.

**Data collection**

*Demographic data*

Disease severity will be calculated using the prognostic indices Acute Physiology and Chronic Health Evaluation (APACHE II) (KNAUS et al., 1985) Simplified Acute Physiology Score (SAPS III) (METNITZ et al., 2005; MORENO et al. 2005), gender, age and admission diagnosis.

*Hemodynamic data*

Heart Rate, Systolic, Diastolic and Mean Blood Pressure, Peripheral Oxygen Saturation, Arterio-Venous Oxygen Difference will be collected. Data will be recorded using a Dixtal DX-2020 monitor before and after the sessions.

*Respiratory Data*

FiO2, respiratory rate and tidal volume will be collected.

*Laboratory Data*

Lactate, Arterial Oxygen Saturation, Arterial Oxygen Pressure, Arterial Carbon Dioxide Pressure and pH through arterial gasometry and Central Venous Oxygen Saturation, Central Venous Oxygen Pressure, Central Venous Carbon Dioxide Pressure and pH through venous gasometry will be analysed before and after the sessions.

*Hospitalization Data*

Length of stay in ICU, days of mechanical ventilation, ICU and hospital mortality, days on vasoactive drugs, corticosteroids and sedation and lower limbs cirtometry will be recorded.

**Statistical analysis**

The data set will be described using tables and figures containing values expressed as mean, standard deviation, median, minimum and maximum values. The generalised linear model will be used to analyse the dependent variables and estimated values of the mean differences of each effect.

The effects are related to the differences of values about the protocols, the sequences and the periods.

The differences will be calculated by subtracting the value at the first protocol time minus the value at the second time. To know if the significance of the differences will use the confidence interval. The p-value will be used to identify whether or not there was an effect of treatment, sequence and period.

The sample size of 31 patients was based on the study by Stefanou et al., 2016 for the variable CD34+, CD133+, CD45- with effect to be detected of 7.3 cells/106, the standard deviation of 10.2, alpha of 5% and power of 80%.
